# Metal chloride cathodes for next-generation rechargeable lithium batteries

**DOI:** 10.1016/j.isci.2024.109557

**Published:** 2024-03-26

**Authors:** Yiming Dai, Shuoqing Zhang, Jiayun Wen, Zhenyou Song, Tengrui Wang, Renyuan Zhang, Xiulin Fan, Wei Luo

**Affiliations:** 1 Institute of New Energy for Vehicles, School of Materials Science and Engineering, Tongji University, Shanghai 201804, China; 2 State Key Laboratory of Silicon and Advanced Semiconductor Materials, School of Materials Science and Engineering, Zhejiang University, Hangzhou 310027, China

**Keywords:** Materials chemistry, Energy materials, Devices

## Abstract

Rechargeable lithium-ion batteries (LIBs) have prospered a rechargeable world, predominantly relying on various metal oxide cathode materials for their abilities to reversibly de-/intercalate lithium-ion, while also serving as lithium sources for batteries. Despite the success of metal oxide, issues including low energy density have raised doubts about their suitability for next-generation lithium batteries. This has sparked interest in metal chlorides, a neglected cathode material family. Metal chlorides show promise with factors like energy density, diffusion coefficient, and compressibility. Unfortunately, challenges like high solubility hamper their utilization. In this review, we highlight the opportunities for metal chlorides in the post-lithium-ion era. Subsequently, we summarize their dissolution challenges. Furthermore, we discuss recent advancements, encompassing liquid-state electrolyte engineering, solid-state electrolytes (SSEs) cooperation, and LiCl-based cathodes. Finally, we provide an outlook on future research directions of metal chlorides, emphasizing electrode fabrication, electrolyte design, the application of SSEs, and the exploration of conversion reactions.

## Graphical abstract

**Figure:**
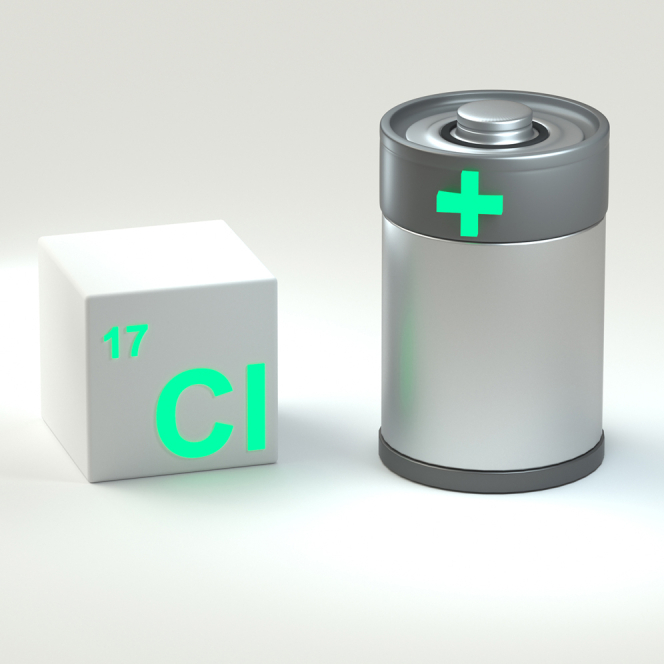

## Introduction

The commercialization of lithium-ion batteries (LIBs) has sparked an era of rechargeable marvel, propelling advancements in portable electronic devices, contributing to the growth of electric transportation and facilitating the creation of the renewable energy storage solutions.^1^^,^^2^ Within the domain of cathode materials for commercial LIBs, metal oxides have asserted their dominance.^3^ The most prevalently used materials among metal oxides include layered oxides (LiCoO_2_ and LiNi_*x*_Co_*y*_Mn_1-*x*-*y*_O_2_), spinel oxides (LiMnO_2_), and polyanion oxides (LiFePO_4_) due to their abilities to reversibly intercalate and deintercalate lithium-ion at considerable operational voltages, while simultaneously serving as lithium sources for batteries.^4^^,^^5^^,^^6^^,^^7^^,^^8^^,^^9^^,^^10^ Although there are many options for oxide cathodes, each of them cannot simultaneously guarantee LIBs achieving high energy density, extended lifetime, and outstanding safety.^11^ As the demand for energy storage continues to escalate, the exploration of potential cathode materials surpass oxides is imperative for fostering batteries beyond the LIBs.^12^^,^^13^^,^^14^ Recently, metal chlorides have attracted increasing attention as cathode materials due to their high energy density, compatibility with solid-state batteries, and cost-effectiveness.^15^^,^^16^^,^^17^^,^^18^^,^^19^

**Figure 1:**
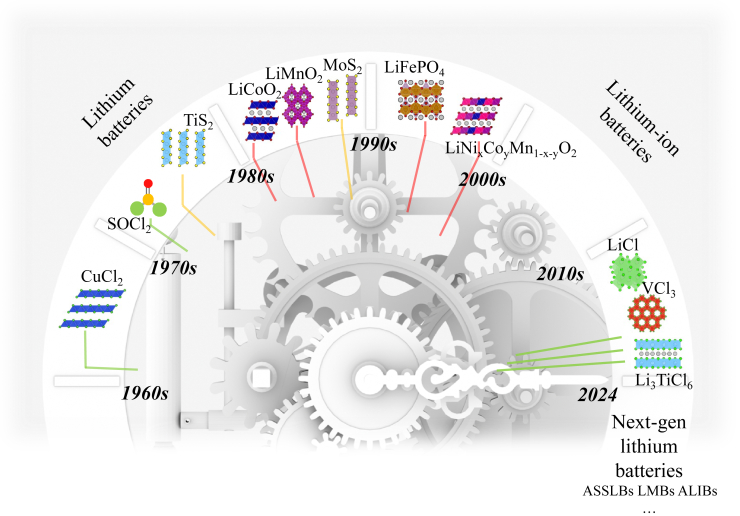
The chronology of cathode materials for various lithium-based batteries

In fact, the idea of applying metal chloride cathodes has been proposed since the 1960s, when lithium batteries were just starting to make their mark, as depicted in the chronology of cathode materials for lithium-based batteries (Figure 1). In 1962, Chilton Jr. and Cook gave a presentation entitled “Lithium Nonaqueous Secondary Batteries.”^4^^,^^20^ In their presentation, they envisioned batteries employing non-aqueous solutions as electrolytes and lithium metal as an anode, paired with metal chlorides such as CuCl_2_, CuCl, and AgCl as cathodes. Among these, CuCl_2_ garnered the most attention due to its cost-effectiveness, theoretical capacity of 400 mAh/g, as well as operational potentials of 3.41 V for the CuCl_2_/CuCl redox couple and 2.74 V for the CuCl/Cu redox couple vs. Li/Li^+^.^21^ Throughout the 1960s, considerable efforts were invested in realizing the applications of CuCl_2_/Li batteries.^22^^,^^23^^,^^24^^,^^25^ However, the dissolution of CuCl_2_ led to subsequent self-discharge issues in batteries, which posed a challenge for its practical application.^26^^,^^27^ Since the successful commercialization of LIBs in the 1990s, metal oxides including LiCoO_2_, LiMnO_2_, LiFePO_4_, and LiNi_x_Co_y_Mn_1-x-y_O_2_ have become the “standard cathodes” for LIBs.^4^ However, in recent years, with the problems related to energy density, longevity, and safety for LIBs, coupled with growing environmental concerns and highlighted supply risks, the development of advanced batteries beyond the traditional ones becomes increasingly important.^28^^,^^29^ Alongside the exploration of novel lithium-based batteries such as lithium metal batteries (LMBs),^30^^,^^31^^,^^32^ all-solid-state lithium batteries (ASSLBs),^33^^,^^34^^,^^35^^,^^36^^,^^37^ and aqueous lithium-ion batteries (ALIBs),^38^^,^^39^ the compatibility of metal oxides with these emerging batteries has been questioned due to reasons including their low energy density, low lithium-ion conductivity, limited compressibility, and high cost.^18^^,^^40^^,^^41^ Following the quest for suitable cathode materials, there is a revival of research in metal chloride-based cathodes nowadays. For example, lithium-free transition metal chlorides like VCl_3_ have been studied to match with lithium metal anodes.^19^^,^^42^ Lithium-containing transition metal chlorides with even higher lithium-ion conductivity and compressibility, such as Li_2_FeCl_4_ and Li_3_TiCl_6_, have been reported as the cathode materials of ASSLBs.^18^^,^^43^^,^^44^ LiCl has also been explored, either directly or indirectly (with *in-situ* generation from SOCl_2_, a catholyte used in primary lithium batteries since the 1970s),^45^ for its potential to greatly lower battery costs and enhance energy density by utilizing anionic redox reactions, thereby eliminating the need for expensive and heavy transition metal cations.^15^^,^^17^^,^^39^ Researchers now may have the opportunity to utilize metal chloride cathodes for advanced battery technologies, leveraging advancements in battery design.

This review aims to introduce the opportunities, challenges and recent advances of metal chloride cathode materials for lithium-based batteries. Firstly, we highlight the opportunities for metal chlorides in the post-lithium-ion era. Secondly, we provide an overview of the challenges posed by their notorious dissolution. Following this, we discuss the recent advancements in chloride cathodes, encompassing electrolyte engineering and the cooperation with solid-state electrolytes (SSEs), along with innovative designs based on LiCl-based cathodes. Lastly, we present perspectives on advancing electrode fabrication technology, screening suitable electrolytes, adopting SSEs, and exploring conversion reactions, all of which constitute potential research directions for metal chloride cathodes.

## Oppotunities

Pursuing higher energy density is the driving force behind battery research. The battery community has set a target of achieving an energy density of 500 Wh kg^−1^.^46^ To reach this goal, designing lithium-based batteries that outperform the traditional LIBs is imperative.^32^ However, the suitability of commercial oxide cathodes in post-lithium-ion batteries is suspectable. For instance, lithium metal batteries, utilizing lithium metal as anodes, unlock the exploration of conversion-type cathodes that could be lithium-poor or even lithium-free.^47^ However, traditional oxide cathodes are predominantly prelithiated, relying on intercalation reactions mechanism, which limits their specific capacities. Additionally, oxide cathodes can hardly work within ASSLBs unless blended with a substantial amount of halide or sulfide SSEs or fused with oxide SSEs through sintering processes.^48^^,^^49^ Worse yet, commercial cathodes employ expensive metals like cobalt and nickel, and require calcination at high-temperature of 500°C–800°C during preparation, leading not only to cost issues but also carbon emissions.^50^^,^^51^ These challenges have generated interest in cathode materials surpass the oxide ones, providing an opportunity for metal chloride to enhance energy density, better collaborate with SSEs, and reduce the cost and carbon footprint of batteries.

Firstly, metal chlorides offer significant potential for conversion reactions due to their excellent operating voltage and specific capacity. A typical conversion reaction involving lithium can be expressed as Equation 1:

(Equation 1) $${TmX}_{m}+nLi\;\leftarrow \to {nLiX}_{m/n}+Tm\;\left(Tm=transition\;metal;\;X=F,Cl,O,S,N,or\;P\right)\text{.}$$

The voltage E of the material’s conversion reaction can be calculated using Equation 2:

(Equation 2) $$E=\left[{\Delta G}_{f}\left({TmX}_{m}\right)–n\Delta G_{f}\left({LiX}_{m/n}\right)\right]/\left(nF\right)\text{,}$$

where ΔG_f_ is the Gibbs free energy of formation (kJ mol^−1^), F is Faraday’s constant (96,485 C mol^−1^), and n is the number of electrons (mol) for this reaction.

At the same time, the specific capacity Q (mAh g^−1^) for materials can be calculated using Equation 3:

(Equation 3) Q=nF/3.6M,

where M is the molecular weight.

**Figure 2:**
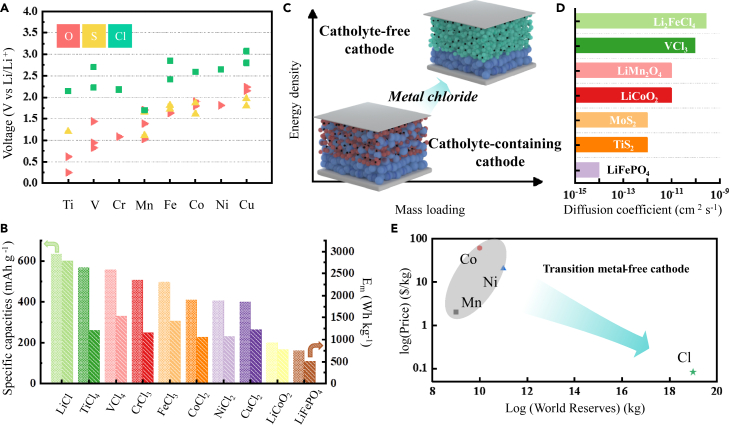
The opportunities of metal chlorides (A) Theoretical operational potential of transition metal oxides, sulfides, and chlorides as conversion-type cathode materials for lithium-based batteries, calculated from Gibbs free energy of formation. (B) A bar chart showing the specific capacity and gravimetric energy density of specific metal chloride cathode materials through conversion reactions, compared to representative lithium-ion battery oxide cathode materials. Grid bars correspond to specific capacity values displayed on the left axis, while hatched bars correspond to gravimetric energy density values shown on the right axis. (C) Comparison of catholyte-containing cathodes and catholyte-free cathodes in ASSLBs. Blue spheres represent SSEs and solid catholytes, black spheres represent conductive carbon, red spheres represent metal oxide cathode materials such as LiFePO_4_ that require catholytes, and green spheres represent metal chloride cathode materials such as Li_3_TiCl_6_ that can operate without catholytes. (D) Chemical diffusion coefficients of some representative metal chlorides, oxides, and sulfides cathodes for lithium-based batteries. (E) Comparison of Cl element with transition metal elements Co, Mn, and Ni in world reserves and prices. Redox reactions involving Cl can enable transition metal-free cathodes, eliminating the reliance on expensive and scarce transition elements.^15^

The thermochemical calculations in Figure 2A show that transition metal chlorides are suitable conversion-responsive cathode materials with higher theoretical operating potentials than oxides and sulfides, mostly above 2 V, in the conversion reaction with lithium. Figure 2B depicts the specific capacities and gravimetric energy densities of certain metal chloride cathode materials in the conversion reaction with lithium. Metal chlorides could surpass that of state-of-the-art metal oxides in energy density if applied as conversion-type cathodes. What’s more, research studies have demonstrated the construction of chloride-ion batteries using electrolytes that is capable of transferring Cl ions.^52^^,^^53^ This opens up additional possibilities for activating conversion reactions through chloride-ion transport as charge carriers.

Furthermore, metal chlorides might be more suitable as cathode materials for post-LIBs, represented by ASSLBs, compared to oxides. For instance, compared to LIBs, ASSLBs impose heightened demands on both lithium-ion diffusion properties and compressibility of cathodes. The first requirement arises from the imperative to augment the mass loading of active materials within the cathode.^49^ As mentioned previously, cathodes of state-of-the-art ASSLBs often contains inert SSEs to construct efficient lithium-ion conduction path. Notably, if the cathode active material possesses significant lithium-ion conductivity, the composite cathode may not require addition of solid electrolyte, thereby significantly improving energy density (Figure 2C).^40^ The second requirement stems from the need to mitigate the cracks of cathode material particles during cycling.^54^^,^^55^^,^^56^ SSEs cannot spontaneously fill the formed cracks like liquids, thus cracks in brittle and non-compressible cathode particles can disrupt the path of lithium-ion migration.^57^^,^^58^^,^^59^ Unfortunately, simultaneously achieving high lithium-ion conductivity and good compressibility is a challenge in oxides. In contrast, transition metal chlorides often exhibit favorable ion conductivity coupled with inherent compressibility. As shown in Figure 2D, the lithium-ion diffusion coefficients of transition metal chlorides such as Li_2_FeCl_4_ and VCl_3_ are in the order of 10^−10^ cm^2^ s^−1^, larger by one to four orders of magnitude compared to representative candidates of oxides and sulfides.^42^^,^^43^ The higher diffusion coefficient aligns with the general observation that the conductivity of SSEs based on metal halides is typically higher than that of chalcogens. In addition to the differences in crystal structure, we believe a more noteworthy factor is the distinction between the two types of anions. Since chloride ions carry only half the charge of oxygen ions, their attraction to lithium ions is weaker, resulting in lower activation energy for the transportation of lithium ions. What is more, the metal chloride cathode Li_3_TiCl_6_ has been documented to exhibit a remarkable compressibility of 86.1% under 350 MPa, akin to metal chloride SSEs.^18^ Those facts imply that metal chloride cathodes can simultaneously address challenges of low mass loading and stress-induced cracks of cathodes during cycling in ASSLBs. Furthermore, apart from their prospects in the field of ASSLBs, the application of metal chlorides extends to other post-Li-ion batteries. For instance, in LMBs, they involve pairing lithium-free layered chloride VCl_3_ with a lithium metal anode,^19^ and in the case of ALIBs, they drive progress through chlorine conversion-intercalation chemistry within the confines of graphite electrodes.^15^^,^^39^

Lastly, metal chlorides could be cost-effective. Metal chlorides such as LiCl, FeCl_2_, and MnCl_2_ are already situated upstream in the chemical industry. They serve as precursor materials for commercial cathodes like LiFePO_4_, LiCoO_2_, and LiNi_x_Co_y_Mn_1-x-y_O_2_. The direct use of chlorides as cathodes can reduce production costs by eliminating the need for a calcination process in the preparation of commercial cathodes. What’s more, LiCl can be utilized as cathode material through anion redox reactions, freeing lithium-based batteries from reliance on expensive transition metals such as Co and Ni (Figure 2E).^15^ Therefore, metal chlorides have the potential to alleviate supply chain challenges and rising costs of commercial oxide cathodes.

## The challenge of dissolution

**Figure 3:**
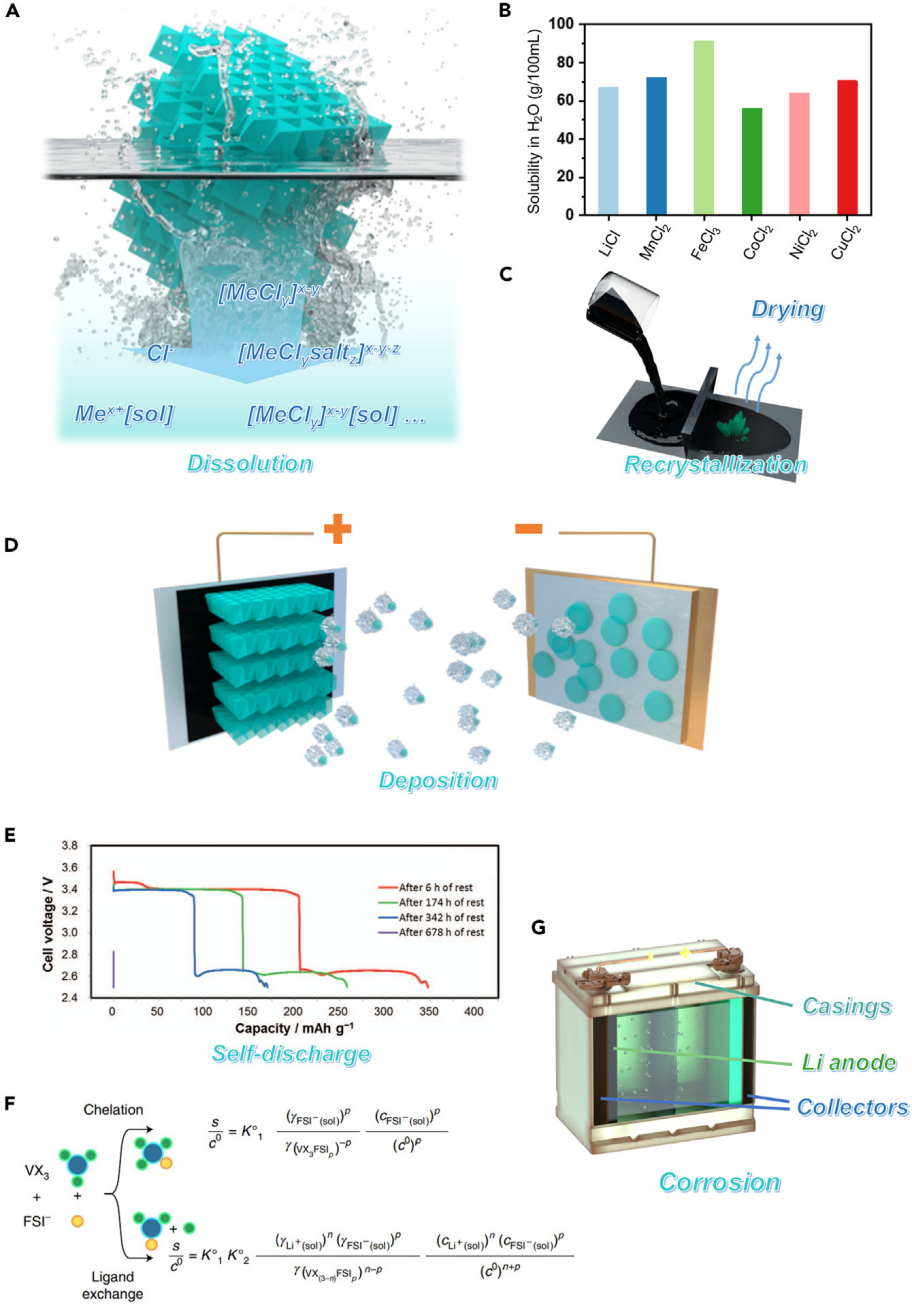
The solubility issues of metal chlorides (A) Scheme illustrating the problem of metal chloride dissolution in electrolytes. (B) A bar chart illustrating the solubility in water of representative metal chlorides (LiCl, MnCl_2_, FeCl_3_, CoCl_2_, NiCl_2_, and CuCl_2_). (C) Scheme depicting the recrystallization of metal chlorides during electrode drying. (D) Scheme illustrating how metal chlorides can dissolve from the cathode, diffuse to the anode through an electric field or concentration gradient, and deposit on the surface of the anode. (E) The severe self-discharge in metal chloride cathodes. The initial discharge capacity of CuCl_2_/Li battery decreases with prolonged resting time. Reproduced with permission from a study by Dobashi et al.^21^ Copyright 2015 IOP Publishing. (F) Expression of vanadium-halide solubility as a function of dissolution based on chelation or ligand exchange mechanism. Experimental evidence supports the ligand exchange mechanism. Reproduced with permission from a study by Dubouis et al.^19^ Copyright 2021 Springer Nature. (G) Scheme illustrating that various metal components within the battery can be corroded by metal chlorides.

While metal chlorides show great potential, they also have some drawbacks, such as electronic insulation and air sensitivity. But perhaps the most vexing challenge might be their notorious dissolution^60^ (Figure 3A). Majority of metal chlorides exhibit high solubility in polar solvents like water, N-Methylpyrrolidone (NMP), and carbonates.^60^^,^^61^ As shown in Figure 3B, the solubility of lithium chloride and certain metal chlorides based on 3 days transition metals exceed 50 mg mL^−1^ in water.^62^ In contrast, the solubility of oxides like LiCoO_2_ and LiFePO_4_ in water are less than 0.5 g mL^−1^. Even worse, when dissolved in electrolytes, anions of supporting salts, solvent molecules, chloride ions, and other components have the potential to complex with metal cations, accompanied by the possibility of independent chloride ion presence, resulting in the intricate dissolution mechanism.^19^ The solubility of chlorides presents at least 4 challenges.

### Cathode fabrication

Current cathode manufacturing processes involve preparing a slurry by dispersing conductive carbon, binder, and active materials in a solvent such as NMP.^63^ This slurry is then coated onto aluminum foil and dried. However, if this traditional cathode manufacturing method is used, the high solubility of metal chlorides in the NMP slurry can result in their dissolution during slurry preparation and recrystallizing into millimeter-sized crystals upon drying (Figure 3C).

### Deposition on the surface of anodes

As shown in Figure 3D, metal chlorides dissolved into the electrolyte from the cathodes might undergo diffusion and migration toward anodes, driven by concentration gradients and electric fields. Subsequently, both chemical substitutions and electrochemical reductions can result in the deposition of metal ion from cathodes onto the surface of anodes. This might result in metal dendrite growth, posing safety concerns.

### Self-discharge

The metal chloride dissolved in the electrolyte cannot participate directly in the electrochemical processes taking place at the positive electrode. Instead, as mentioned earlier, it reacts with the negative electrode chemically. Consequently, residual self-discharge is expected to occur in the battery. As demonstrated by Dobashi et al., the CuCl_2_/Li batteries would fail after hundred hours of left idle if unoptimized electrolytes are used (Figure 3E).^21^

### Corrosion

Aluminum foil and copper foil are commonly used as current collectors for electrodes, while steel and aluminum serve as battery casings. Unfortunately, most transition metal chlorides are potent Lewis acids and thermodynamically favorable to undergo substitution reactions with these materials.^64^ Even worse, experiments by Nicolas Dubouis et al. demonstrated that the dissolution of metal chlorides in the electrolyte may proceed via a ligand exchange mechanism, resulting in the presence of free Cl^−^ ions in the electrolyte (Figure 3F).^19^ These free Cl^−^ ions, due to their small ionic radius, can penetrate the passive film on metal surfaces, thereby accelerating the kinetics of substitution reactions, which can cause severe corrosion issues.^65^ For instance, promising cathode candidates like FeCl_3_ can corrode aluminum, copper, and even stainless steel. As shown in Figure 3G, all metallic components in the batteries are susceptible to reactions with FeCl_3_. This not only leads to rapid degradation of battery performance but also poses a risk of leakage.

Based on the aforementioned reasons, circumventing the dissolution of metal chlorides is essential for the advancement of metal chloride cathodes. Therefore, while researching methods to mitigate the dissolution of metal chlorides, it is crucial to delve deeper into the underlying mechanisms. Additionally, during battery research and testing, it is important to mitigate the impact of the high reactivity of metal chlorides through the selection of inert current collectors and battery casings.

## Recent advance

In recent years, efforts have been made to address the dissolution challenges of metal chloride cathodes, rendering them a promising prospect. Rational electrolyte design has been demonstrated to mitigate the dissolution of metal chlorides.^19^^,^^21^ Within ASSLBs, metal chloride cathodes exhibit commendable reversibility, primarily attributed to the prevention of dissolution of soluble active materials.^42^ Moreover, upon the inhibition of dissolution, the reversible redox reactions of chloride ions in LiCl are unlocked,^39^ offering the potential for cathode materials to deliver capacity without reliance on transition metals.

### Electrolyte engineering

**Figure 4:**
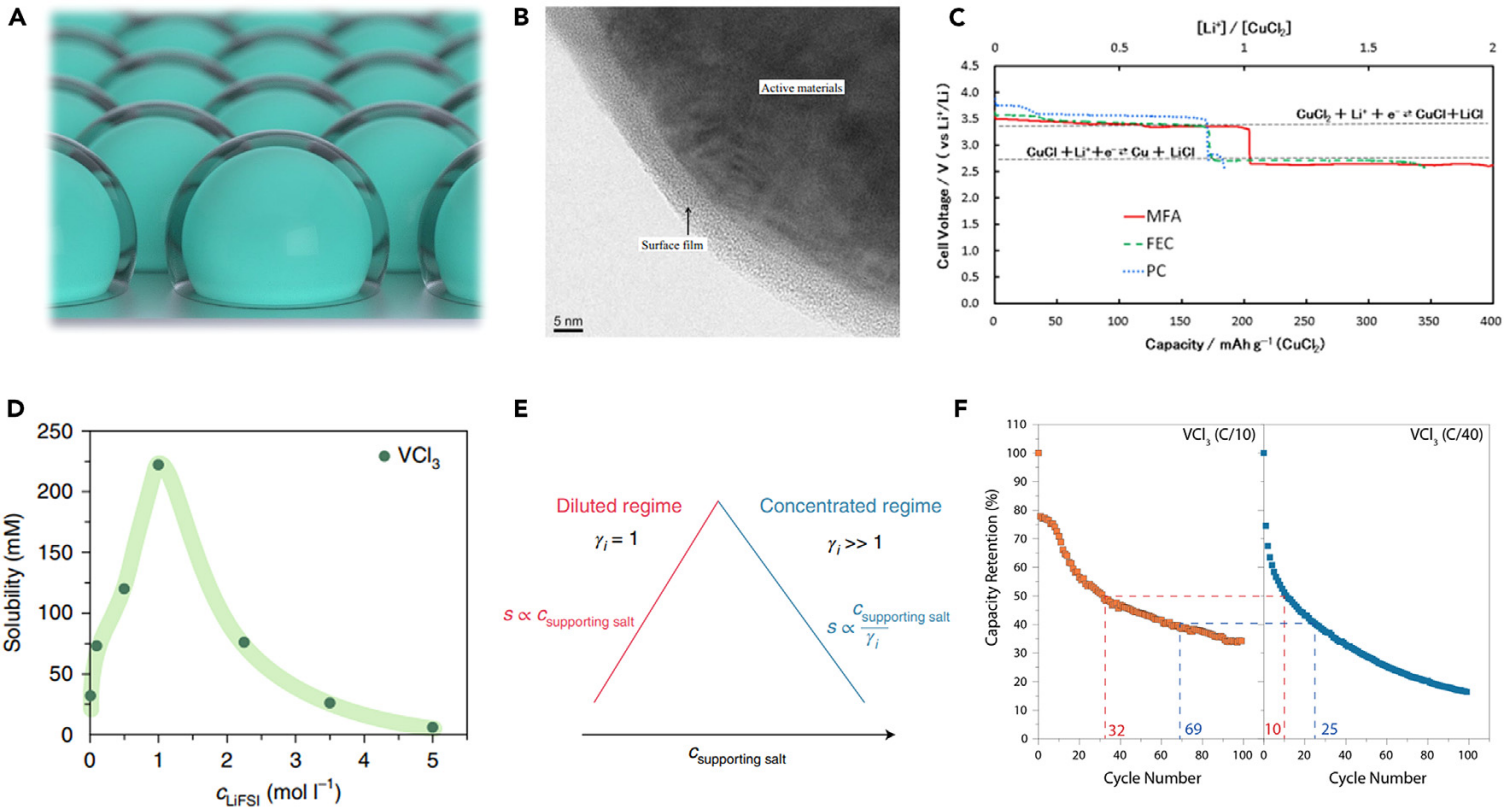
Electrolyte engineering for suppressing the dissolution of metal chlorides (A) Illustration of stabilizing metal chloride cathodes through stable CEI films. (B) The CEI film of CuCl_2_ observed by transmission electron microscope (TEM) after 4-week (672 h) immersion in 2.2 M LiPF_6_/MFA electrolyte. Reproduced with permission from a study by Hashizaki et al.^68^ Copyright 2019 IOP Publishing. (C) Discharge characteristics of CuCl_2_ electrode in different electrolytes. Reproduced with permission from a study by Hashizaki et al.^68^ Copyright 2019 IOP Publishing. (D) Variation of VCl_3_ solubility with LiFSI/DMC electrolytes as a function of the LiFSI concentration (c_LiFSI_). Reproduced with permission from a study by Dubouis et al.^19^ Copyright 2021 Springer Nature. (E) Explanation for the evolution of VCl_3_ solubility. The thermodynamics laws govern the transition-metal solubility as a function of the supporting salt concentration (c_supporting__salt_). Reproduced with permission from a study by Dubouis et al.^19^ Copyright 2021 Springer Nature. (F) Discharge capacity retention observed for VCl_3_ cycled at either C/10 (orange) or C/40 (blue) rates. Reproduced with permission from a study by Dubouis et al.^19^ Copyright 2021 Springer Nature.

The electrolytes typically consist of organic solvents and lithium salts in lithium batteries.^66^ The composition and concentration of the electrolyte greatly influence the nature of the cathode-electrolyte interphase (CEI) and the solubility of electrode materials.^67^ To regulate the solubility of metal chlorides and enhance performance, electrolyte engineering might be one of the most effective and economical avenues. A high-quality CEI layer with integrity, stability, and uniformity can effectively inhibit the dissolution of metal chlorides and extend battery life (Figure 4A). Moreover, recent research advancements also provided possibilities for modulating the thermodynamic processes of dissolution through electrolyte engineering.

Ogumi’s group reported the application of CuCl_2_ as a conversion-type cathode by utilizing fluorinated electrolytes.^21^^,^^68^^,^^69^ Their particular electrolyte formulation of methyl difluoroacetate (MFA) as the solvent and LiPF_6_ as the lithium salt successfully mitigated the dissolution of the CuCl_2_ cathode. This improvement could be attributed to two factors. Firstly, the fluorinated solvent (e.g., MFA) had characteristics like low dielectric constant and limited CuCl_2_ solubility. Secondly, the presence of LiPF_6_ enabled the formation of an obstructive CEI on the surface of cathode (Figure 4B). These two points acted synergistically to effectively impede the dissolution of CuCl_2_. As illustrated in Figure 4C, the discharge capacity of the assembled battery was almost close to the theoretical capacity (400 mAh g^−1^), while the two-electron redox reaction was clearly visible. Compared to the batteries with non-optimized electrolytes, the self-discharge of the batteries with fluorinated electrolyte was alleviated and the rechargeability was improved.

Apart from the modulation of CEI, it has also been reported that the dissolution of metal chlorides can be suppressed at the thermodynamic level.^19^ Recently, Dubouis et al. reported the application of superconcentrated electrolytes in preventing the dissolution of the VCl_3_ and facilitating the insertion of lithium ions into their structure. Firstly, they experimentally demonstrated that the solubility of VCl_3_ in dimethyl carbonate (DMC) gradually increased with the addition of the supporting salt lithium bis(fluorosulfonyl) imide (LiFSI) (Figure 4D). The solubility of VCl_3_ reached its highest value when the concentration of LiFSI reached 1 M and then decreased with the increase of LiFSI concentration. Furthermore, the authors also revealed that the low solubility of the VCl_3_ in high-concentration electrolytes depended on the nature of the halide ligand and the activity coefficient of the vanadium complex in solution (Figure 4E). Although the capacity decay of such cells was still severe (Figure 4F), their work demonstrated the possibility of tailoring the dissolution through thermodynamic rather than kinetic effects.

### All-solid-state lithium batteries

The development of ASSLBs using SSEs has brought new opportunities for metal chloride cathodes. Owing to potentially high safety and high energy density characteristics, ASSLBs are viewed as promising next-generation lithium-based batteries.^34^^,^^35^ What is more, due to the solid inorganic electrolytes allowing only the transport of lithium-ion, they might be intrinsically beneficial in preventing the dissolution of soluble components from the cathode.^61^^,^^70^

**Figure 5:**
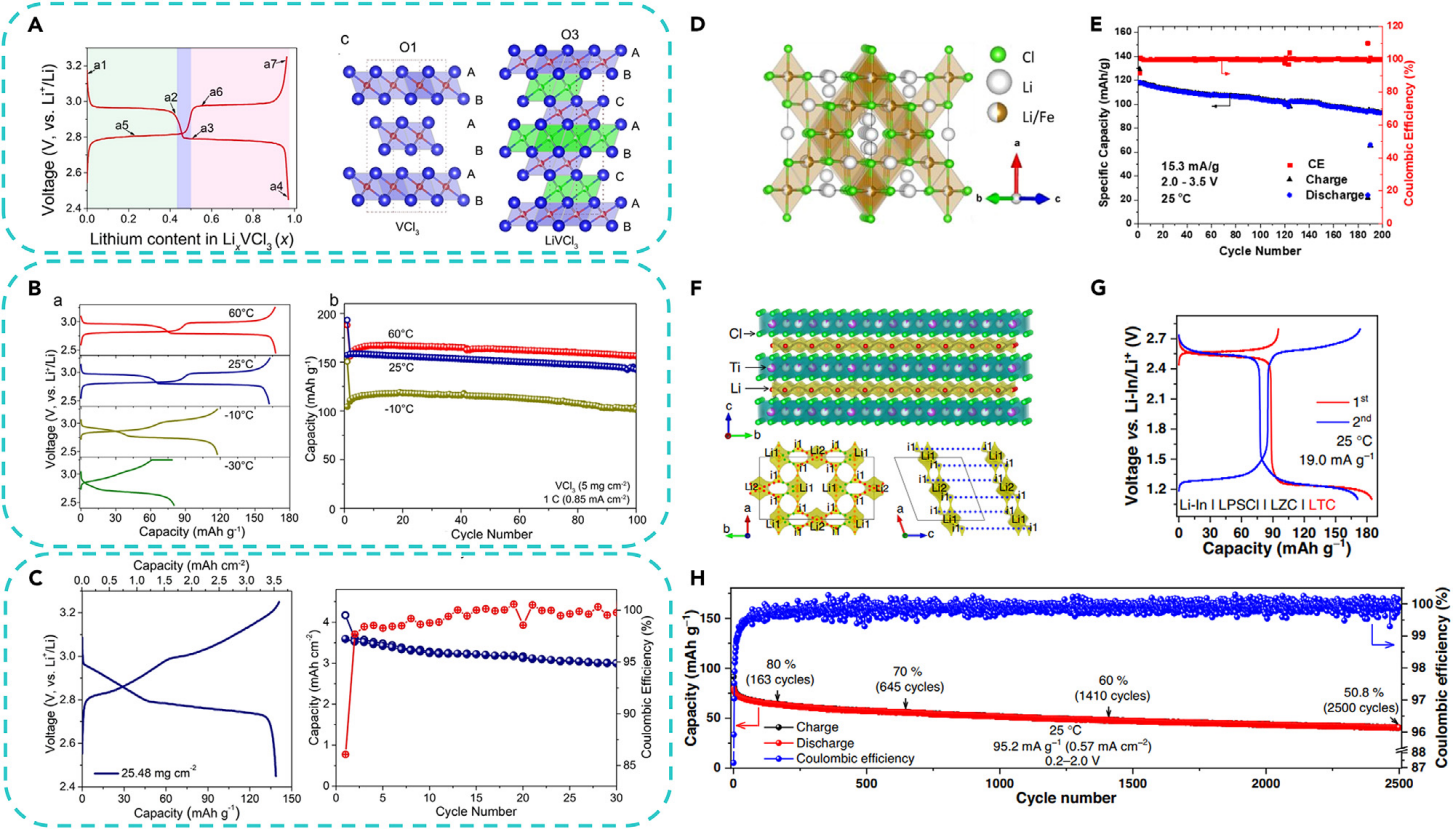
Application of metal chlorides as cathodes for ASSLBs (A) Typical discharge/charge curves of VCl_3_-Li_3_InCl_6_-C cathode for ASSLBs. VCl_3_ (the layered O1 structure) and LiVCl_3_ (the layered O3 structure). Reproduced with permission from a study by Liang et al.^42^ Copyright 2023 Wiley-VCH. (B) Galvanostatic discharge/charge curves and cycling performance of the VCl_3_-Li_3_InCl_6_-C cathode at different temperatures. Reproduced with permission from a study by Liang et al.^42^ Copyright 2023 Wiley-VCH. (C) Galvanostatic discharge/charge curves and cycling performance of the VCl_3_-Li_3_InCl_6_-C cathode with high mass loadings (25.48 mg cm^−2^). Reproduced with permission from a study by Liang et al.^42^ Copyright 2023 Wiley-VCH. (D) Crystal structure of the cubic LFC. Reproduced with permission from a study by Vinado.^71^ Copyright 2019 Carolina Vinado. (E) Cycling performance and Coulombic efficiency of the LFC/Li_3_InCl_6_/Li-In cell. Reproduced with permission from a study by Vinado.^71^ Copyright 2019 Carolina Vinado. (F) The structural model of LTC superimposed with the lithium-ion potential map.^18^ Reproduced with permission from a study by Wang et al.^18^ Copyright 2023 Springer Nature. (G) The reversible Ti^2+^/Ti^3+^ and Ti^3+^/Ti^4+^ redox couples. Reproduced with permission from a study by Wang et al.^18^ Copyright 2023 Springer Nature. (H) Long-term cycling performance of the single-LTC cells. Reproduced with permission from a study by Wang et al.^18^ Copyright 2023 Springer Nature.

Recently, Sun’ group proposed halide-based ASSLBs with VCl_3_ cathodes and Li_3_InCl_6_ SSEs. VCl_3_ possessed the O1 layered structure with an R-3m space group, while it turned into O1-type structure and R-3 space group after being lithiated into LiVCl_3_ (Figure 5A).^42^ It delivered a reversible capacity of 203.8 mAh g^−1^ at 0.1 C, and a high-rate capability of 152.4 mAh g^−1^ at 6 C as well as a long cycle life of over 200 cycles with 85% capacity retention. In addition, the system also showed a good performance at extreme temperatures of −30°C and 60°C (Figure 5B), and an ultra-high loading density of up to 25 mg cm^−2^ (Figure 5C). Compared to the rapid capacity decay of VCl_3_ in liquid-state batteries, ASSLBs exhibited greater potential for maintaining capacity retention. Moreover, they also experimentally proved that the compatibility between metal chloride cathodes and metal chloride solid electrolytes is considered to be good. It was demonstrated that VCl_3_ and Li_3_InCl_6_ or Li_3_HoCl_6_ solid electrolytes could form composite cathodes and achieve stable cycling. By employing chloride-based solid electrolytes, there is potential to manufacture advanced all-solid-state batteries.

In addition to lithium-free transition metal chlorides, there have also been reports and patents on lithium-containing transition metal compounds, such as lithium transition metal tetrachlorides (Li_2_TMCl_4_) and lithium transition metal hexachlorides (Li_3_TMCl_6_).^18^^,^^43^ Li_2_TMCl_4_, in particular, received early attention, possibly due to its higher theoretical capacity. Research has focused on compounds like Li_2_FeCl_4_ (LFC), Li_2_CrCl_4_, Li_2_MnCl_4_, Li_2_CoCl_4_, etc., among which LFC has garnered the most attention.^72^ The LFC exhibited a cubic crystalline structure with a space group $Fd\bar{3}m$. It is structured with 3D channels facilitating the rapid lithium-ion diffusion, as illustrated in Figure 5D. The LFC adopted a face-centered inverse spinel configuration, with half of the lithium occupying tetrahedral positions and the other half along with iron occupying octahedral positions. Notably, the diffusion coefficient of LFC (2.8 × 10^−10^ cm^2^ s^−1^) was more than four orders of magnitude higher than that of LiFePO_4_(10^−14^ cm^2^ s^−1^), and the operational potential lied around 3.2 V. Moreover, assuming reversible extraction of approximately 1.2 Li^+^ ions per chemical formula, the theoretical specific capacity of LFC could reach 153 mAh g^−1^. The Figure 5E shows the cycling performance of the LFC/Li_3_YCl_6_/In-Li cell at 0.1 C. The capacity retention was considerable, as it maintained over 80% (∼105 mAh g^−1^) of its initial capacity (∼130 mAh g^−1^) after 180 cycles. Given the favorable electrochemical performance of LFC, along with the abundance and cost-effectiveness of iron elements, the utilization of LFC as a cathode material could potentially be applied in low-cost ASSLBs.

In addition to the Li_2_TMCl_4_, Li_3_TMCl_6_ have also been preliminarily studied. Recently, Li_3_TiCl_6_ (LTC), which can be used as a novel cathode material for ASSLBs, was proposed by Wang et al.^18^ As schematically illustrated in Figure 5F, the LTC materials seem isostructural with the Li_3_InCl_6_, showing a layered structure and C2/m space group. Interestingly, bond valence site energy calculation suggested that the lithium-ion transport may occur both within and between the a-b planes, which gave LTC more lithium-ion migration directions than the layered oxides like LiCoO_2_. The LTC material had high ionic conductivity (1.04 mS cm^−1^ at 25°C), good compressibility (86.1% under 350 MPa), and reversible redox reactions of Ti^3+^/Ti^4+^ and Ti^2+^/Ti^3+^ (∼185 mAh g^−1^) at room temperature (Figure 5G). The LTC material could be used as a cathode with a high mass loading of 95 wt % without the need to add any solid electrolyte. The Li-In | Li_6_PS_5_Cl | Li_3_ZrCl_6_ | LTC cell based on Ti^2+^/Ti^3+^ and Ti^3+^/Ti^4+^ redox couples delivered an initial discharge capacity of 184.5 mAh g^−1^ and a capacity retention of 60.2% after 100 cycles at 25°C. Furthermore, the LTC material could be used as electrolyte, anode and cathode simultaneously in a single-material cell. As shown in Figure 5H, such a single-LTC cell exhibited a decent cycle life (capacity retention above 80% and 60% after 163 and 1,410 cycles, at 95.2 mA g^−1^ at 25°C, respectively).

### LiCl-based cathodes

The utilization of LiCl as cathode materials is a novel design in recent years.^15^^,^^16^^,^^17^^,^^39^^,^^45^^,^^73^^,^^74^^,^^75^^,^^76^ LiCl possesses an impressive theoretical specific capacity of 632 mAh g^−1^ and an operational potential of 4.4 V,^74^ which yields an unparalleled gravimetric energy density of 2,780.8 Wh kg^−1^. Furthermore, it provides capacity through anion redox reactions, eliminating the need for transition metal elements such as Co and Ni and potentially making cathodes more cost-effective.

**Figure 6:**
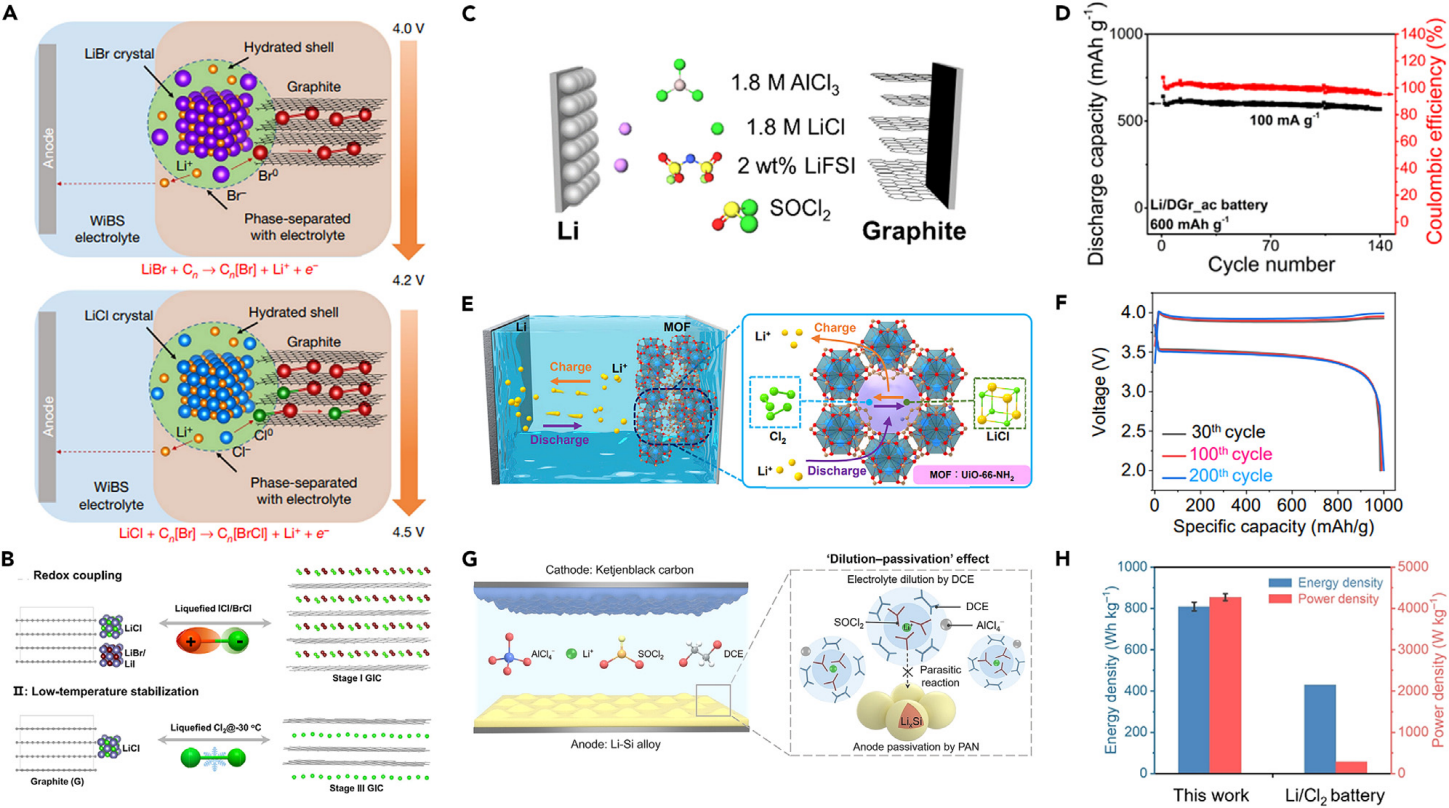
LiCl-based cathodes for lithium-based batteries (A) Schematic of the conversion-intercalation mechanism of the LiCl-LiBr-graphite composite cathode. Reproduced with permission from a study by Yang et al.^39^ Copyright 2019 Springer Nature. (B) Schematic of liquefying Cl by forming ICl and BrCl interhalogens or reducing the temperature to −30°C to create stable graphite intercalated compounds (GICs). Reproduced with permission from a study by Xu et al.^15^ Copyright 2022 Elsevier. (C) Schematic of a rechargeable Li/Cl_2_ battery utilizing catholyte containing SOCl_2_ as the active material, Li as the anode, and defective graphite or CO_2_-activated graphite as the cathode. Reproduced with permission from a study by Zhu et al.^73^ Copyright 2022 American Chemical Society. (D) Cycling performance of Li/Cl_2_ battery with a Li/DGr_ac cell using CO_2_-activated graphite as the positive electrode. Reproduced with permission from a study by Zhu et al.^73^ Copyright 2022 American Chemical Society. (E) Schematic of the Li-Cl_2_@MOF (UiO-66-NH2) cell and the cathode Cl_2_/LiCl reaction mechanism. Reproduced with permission from a study by Xu et al.^76^ Copyright 2023 Elsevier. (F) Corresponding voltage profiles of the Li-Cl_2_@MOF cell in different cycles. Reproduced with permission from a study by Xu et al.^76^ Copyright 2023 Elsevier. (G) Schematic of the Li-Si/Cl_2_ battery. DCE and PAN represent 1,2-dichloroethane and polyacrylonitrile, respectively. Reproduced with permission from a study by Yuan et al.^75^ Copyright 2023 Wiley-VCH. (H) Comparison of the energy and power densities between Li-Si/Cl_2_ and Li/Cl_2_ batteries. Reproduced with permission from a study by Yuan et al.^75^ Copyright 2023 Wiley-VCH.

In 2019, Yang et al. proposed a novel intercalation-conversion chemistry for ALIBs that employed a composite material of LiCl, LiBr, and graphite as the cathode.^39^ As shown in Figure 6A, during the charging process, LiBr and LiCl were successively converted into Br^0^ and Cl^0^, which are embedded into the graphite interlayer through a biphasic highly concentrated aqueous electrolyte and stabilized in the form of a solid graphite intercalation compound (GIC). They demonstrated that this halogen conversion-intercalation cathode could achieve a remarkably high reversible capacity of 243 mAh g^−1^ (considering the total electrode weight) at an average potential of 4.2 V versus Li/Li^+^, resulting in an energy density of 970 Wh kg^−1^ for the cathode. By coupling this cathode with a passivated graphite anode, a 4-volt-class ALIB full cell with an energy density of 460 Wh kg^−1^ was fabricated (considering the total weight of both electrodes). The configuration maintained a stable capacity over 150 charge-discharge cycles with a Coulombic efficiency approximately up to 100%. This innovative design integrated the advantageous capacities of conversion reactions, the excellent reversibility of intercalation mechanisms, and the enhanced safety characteristic of aqueous batteries.

Recently, Xu et al. have further advanced the study of the intercalation-conversion chemistry of lithium halide-graphite cathodes by directly combining the lithium halide-graphite cathode with a lithium metal anode using a quasi-ionic liquid electrolyte.^15^ This advancement was based on two key insights: (1) to liquefy halogens or interhalogens by forming interhalogen compounds with varying electronegativity or by reducing temperature to facilitate the reversible intercalation of halogens into graphite, as shown in Figure 6B; and (2) to design a quasi-ionic liquid electrolyte with high anodic stability and low solubility for halogens and halides, thereby preventing electrolyte degradation and halogen loss. They demonstrated that the LiCl-LiBr-graphite cathode achieved notable reversible capacities (up to 250 mAh g^−1^), impressive energy densities (up to 770 Wh kg^−1^, LiCl-LiBr-graphite (2.5 mAh/cm^2^)||Li (20 μm), pouch cells), and extended cycle lifetimes (retaining 80% capacity after 400 cycles) across a range of temperatures (from −30°C to 25°C).

In addition to the direct use of LiCl as a cathode material, another option is the *in-situ* formation of LiCl-host composites from the active components such as SOCl_2_ in the catholyte during the initial discharge process.^17^^,^^45^^,^^73^^,^^75^^,^^76^ Interestingly, SOCl_2_ has been developed as a catholyte for primary batteries since 1970s,^77^^,^^78^ boasting high capacity and working voltage, yet it has long been reported to lack rechargeability. Nowadays, after 50 years, its rechargeability is achieved.

Zhu et al. proposed a rechargeable Li/Cl_2_ battery utilizing amorphous carbon nanospheres (aCNS) as the cathode and AlCl_3_ in SOCl_2_ as the starting electrolyte.^17^ The battery operated through redox reactions between Cl_2_/Cl^−^ inside the micropores of the carbon cathode and Li/Li^+^ redox on the lithium metal anode, achieving a high capacity of 3,309 mAh g^−1^. The key to achieving rechargeability lied in the highly reversible liquid/solid-gas conversion at the cathode and the stable metal-electrolyte interface at the anode. The successful development of rechargeable Li-Cl_2_ systems showed a pathway of high-energy rechargeable battery utilizing reversible anionic redox of LiCl directly.

Along this path, more electrode materials are being gradually explored. Zhu and his colleagues further utilized CO_2_-activated defective graphite (DGr_ac) as a cathode material (Figure 6C), which had an increased surface area and pore volume.^73^ When operating at a capacity of 600 mAh g^−1^ and a current of 100 mA g^−1^, the battery was cycled more than 140 times. (Figure 6D). Xu et al. reported NH_2_-functionalized MOF (UiO66-NH_2_) as a cathode material, where the NH_2_ functional group acting as a Lewis base to enhance the stability of the positive electrode by interacting with the Lewis acids LiCl and Cl_2_ (Figure 6E).^76^ As shown in Figure 6F, high stability for 200 cycles were achieved under a specific capacity of 1,000 mAh g^−1^ at room temperature. Yuan et al. also reported the Ketjen black carbon cathode material and paired it with a Li-Si alloy anode (Figure 6G).^75^ By removing excess lithium from the anode, they achieved the first rechargeable Cl_2_ full battery with remarkable energy and power densities of 809 Wh kg^−1^ and 4,277 W kg^−1^, respectively (Figure 6H).

## Conclusion and perspectives

Six decades after the initial proposal of the CuCl_2_/Li battery in 1962, metal chloride cathodes have regained the interest of researchers for the next generation of rechargeable lithium-based batteries. However, they are still in the early stages of exploration and far from practical application. Many properties, such as processability and cycling stability, require further optimization. Based on our understanding, we offer several perspectives for the potential research directions of these materials.

### Electrode fabrication technology

**Figure 7:**
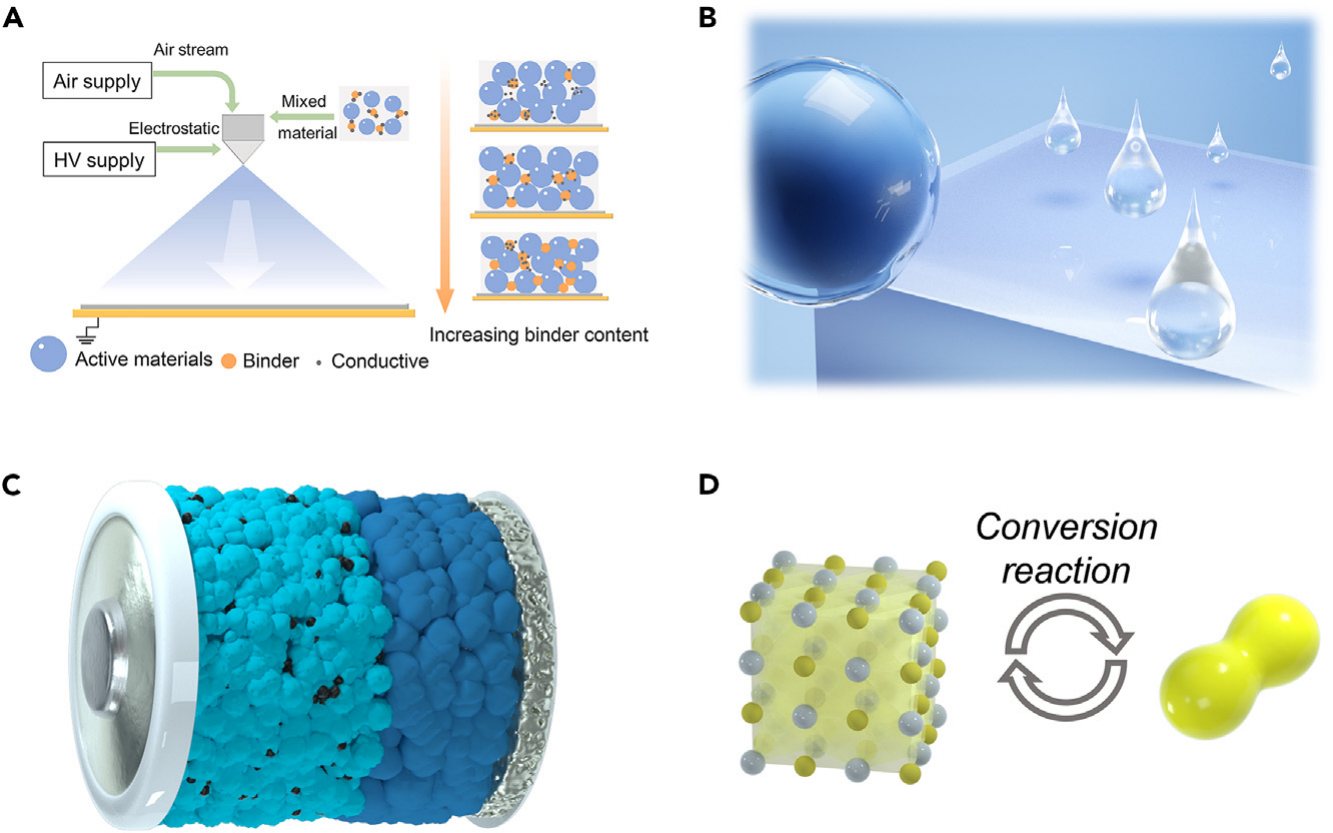
Schematics showing the potential research directions of metal chloride cathode materials (A) Schematic of solvent-free electrode manufacturing. Reproduced with permission from a study by Zhen et al.^79^ Copyright 2021 Elsevier. (B) Schematic of suitable electrolyte systems to suppress the dissolution of metal chlorides and support their operation as cathodes. (C) Schematic of assembling all-solid-state batteries with metal chloride cathodes. The green particles represent metal chloride cathodes, the blue particles represent SSEs, and the black particles represent conductive carbon. (D) Schematic of the aggressive conversion between halogens and lithium halides.

To circumvent the recrystallization of electrodes during drying process, it is necessary to develop electrode fabrication methods that go beyond traditional coating processes. Therefore, the exploration of approaches such as non-polar-solvent and solvent-free electrode fabrication is crucial. Some works have demonstrated the feasibility of using non-polar solvents or even solvent-free methods to fabricate solid electrolyte membranes based on metal chlorides (Figure 7A).^79^^,^^80^^,^^81^^,^^82^^,^^83^ These novel techniques for producing metal chloride electrodes should be further explored.

### Appropriate electrolytes

In recent years, new electrolyte concepts such as high-concentration electrolytes, localized high-concentration electrolytes, fluorinated electrolytes, and weakly solvating electrolytes have been developed.^84^^,^^85^^,^^86^^,^^87^^,^^88^^,^^89^^,^^90^^,^^91^^,^^92^ They have been well validated in the study of other soluble cathodes such as sulfur and iodine, which effectively inhibit the dissolution of this soluble cathode.^84^^,^^85^^,^^93^^,^^94^^,^^95^^,^^96^^,^^97^ By applying these innovative electrolyte concepts, we are poised to design electrolytes capable of supporting metal chloride cathodes (Figure 7B).

### ASSLBs

Since the solid inorganic electrolytes allow only the transport of lithium ions, they might inherently prevent the dissolution of soluble components from the cathode.^70^^,^^98^^,^^99^^,^^100^^,^^101^ This points to a new avenue for addressing the dissolution issue of metal chlorides. Moreover, while SSEs resolve the dissolution problem of metal chlorides, metal chloride cathodes simultaneously address issues such as low active material loading and stress-induced failures during cycling in solid-state batteries.^18^ Therefore, we believe that metal chloride cathodes and SSEs might present mutual opportunities (Figure 7C). Further exploration should be conducted on metal chlorides with various structures and compositions. Additionally, the potential research direction of using metal chlorides as capacity-providing catholytes in combination with oxide cathodes should also be considered.

### Conversion reactions

To achieve a new milestone of >500 Wh kg^−1^, it is worth challenging the use of electrode materials with certain conversion-reaction characteristics.^41^^,^^102^ Excitingly, Chen et al. suggested that, similar to oxygen ions, chloride ions can undergo anion redox reactions, providing additional capacity.^103^ Meanwhile, chlorine exhibits high abundance in nature, with its abundance in seawater reaching 19,400 mg L^−1^, while fluorine is 1.3 mg L^−1^, bromine is 67 mg L^−1^, and iodine is 0.06 mg L^−1^.^104^ This indicates the potential of chlorine for developing low-cost, large-scale energy storage technologies. More interestingly, in the case of metal chlorides, both chloride ions and lithium ions can serve as charge carriers during conversion reaction.^19^^,^^21^^,^^52^^,^^53^^,^^68^ However, the distinction between the two lacks sufficient research. Additionally, the aggressive conversion between halogens and lithium halides holds the promise of higher energy densities,^17^^,^^39^ but unfortunately also presents greater challenges in terms of safety and reversibility (Figure 7D). Therefore, developing suitable strategies to make the reactions of these highly reactive halogens controllable could emerge as a pivotal focus.

In pursuit of these research directions, we can look forward to the continued development of metal chloride cathodes, potentially paving the way for high-energy, longevous, and cost-effective lithium-based batteries in the future.

In summary, metal-chloride cathode materials are showing robust vitality. Despite the numerous challenges posed by the high solubility of metal chlorides, which impact electrode manufacturing and electrolyte compatibility, as well as the need for further optimization of their electrochemical performance, researches have shown us that with rational design, metal chloride cathodes have the potential to make the next-generation lithium-based batteries significantly higher in energy density, more longevous, and more cost-effective. Considering the new insights, materials, and characterization techniques we have today, we might find ourselves at an intriguing turning point, capable of unlocking the potential of metal chlorides that have remained dormant for years and developing groundbreaking energy storage solutions.
